# Case Report: Certolizumab pegol as a potential rescue option for severe erythema multiforme associated with pustular psoriasis during pregnancy

**DOI:** 10.3389/fmed.2026.1933101

**Published:** 2026-10-13

**Authors:** Yao Lu, Sha Lai, Qiuqin Ji

**Affiliations:** Department of Dermatology, Chengdu Women’s and Children’s Central Hospital, School of Medicine, University of Electronic Science and Technology of China, Chengdu, China

**Keywords:** anti-TNF therapy, certolizumab pegol, erythema multiforme, pregnancy, pustular psoriasis presenting during pregnancy

## Abstract

**Background:**

Severe erythema multiforme (EM) and pustular psoriasis presenting during pregnancy (PPP) are rare inflammatory dermatoses that may adversely affect maternal and fetal outcomes. Their coexistence during pregnancy is exceptionally uncommon, and therapeutic decisions are challenging because of limited evidence and concerns regarding fetal safety.

**Case presentation:**

A 37-year-old woman at 30 weeks of gestation presented with rapidly progressive targetoid erythema, papulovesicular lesions, blisters, pustules, acral edema, and mild oral mucosal erosion. Severe EM was initially suspected based on the presence of targetoid lesions and mucosal involvement. During hospitalization, the development of sterile pustules and pustular plaques suggested concomitant PPP. Histopathological examination demonstrated interface dermatitis with focal epidermal necrosis in the arm lesion, consistent with EM, and intraepidermal pustule formation with neutrophilic infiltration in the abdominal lesion, supporting PPP. Potential EM triggers were investigated. HSV-I/II IgM was positive, whereas Mycoplasma pneumoniae testing was negative; however, no definitive infectious trigger was identified. Hydroxychloroquine, initiated approximately 2 weeks before disease onset by an external obstetrician, was discontinued because drug-associated EM was considered possible, although causality could not be confirmed. Despite treatment with intravenous methylprednisolone, intravenous immunoglobulin (IVIG), and corticosteroid pulse therapy, the disease continued to progress. Histopathological examination supported the diagnoses of EM and PPP. Genetic analysis revealed wild-type IL36RN and a heterozygous IL36G c.404T > C variant of uncertain significance.

**Intervention and outcome:**

After infection was excluded and informed consent was obtained, a single dose of certolizumab pegol 400 mg was administered subcutaneously during the early corticosteroid tapering phase following methylprednisolone pulse therapy Cutaneous lesions showed marked improvement within 72 h after certolizumab administration. Corticosteroids were successfully tapered. The patient delivered a healthy male infant vaginally at 39 weeks and 1 day of gestation. No maternal or neonatal adverse events were observed, and remission was maintained during one year of postpartum follow-up.

**Conclusion:**

This case highlights the diagnostic complexity and therapeutic challenges of concurrent severe EM and PPP during pregnancy. Although the contribution of previous corticosteroid and IVIG therapy cannot be completely excluded, the temporal association between certolizumab pegol administration and clinical improvement suggests that TNF-α blockade may represent a potential option. Further clinical experience and registry-based studies are required to clarify its efficacy and optimal clinical application.

## Introduction

Pregnancy-associated inflammatory dermatoses represent a therapeutic challenge because clinicians must balance rapid maternal disease control with fetal safety considerations. Immune and endocrine adaptations during pregnancy may alter inflammatory pathways and influence the clinical course of pre-existing or newly developed skin diseases.

Erythema multiforme (EM) is an immune-mediated mucocutaneous disorder characterized by acute-onset targetoid lesions and variable mucosal involvement. Infectious agents and medications are recognized triggers, and severe presentations with extensive blistering or mucosal involvement require careful differentiation from Stevens–Johnson syndrome/toxic epidermal necrolysis and other severe cutaneous adverse reactions (1). Although EM is generally self-limited, severe or progressive disease during pregnancy may require systemic immunomodulatory therapy.

Pustular psoriasis presenting during pregnancy (PPP), historically referred to as impetigo herpetiformis, is a rare and potentially life-threatening inflammatory dermatosis that is increasingly considered part of the generalized pustular psoriasis spectrum (2, 3). The disease may be associated with extensive sterile pustulation, systemic inflammation, electrolyte imbalance, placental dysfunction, preterm delivery, fetal growth restriction, and fetal loss. Systemic corticosteroids and cyclosporine remain commonly used therapeutic options; however, refractory cases continue to represent a significant clinical challenge, particularly when prolonged immunosuppression is undesirable during pregnancy (3, 4).

Certolizumab pegol is a pegylated, humanized anti-tumor necrosis factor-alpha (TNF-α) Fab fragment that lacks an Fc region, thereby limiting Fc receptor-mediated placental transfer. Pharmacokinetic studies have demonstrated minimal fetal exposure during late pregnancy, supporting its potential role as a biologic option when anti-inflammatory therapy is required in pregnant patients (5). However, clinical experience with certolizumab pegol in severe pregnancy-associated inflammatory dermatoses remains limited.

## Case report

### Patient information and initial presentation

A 37-year-old pregnant woman (gravida 4, para 0, body weight, 59 kg) at 30 weeks of gestation was admitted on January 28, 2025, because of a 2-day history of rapidly progressive generalized erythematous eruptions, papules, and blisters.

Her pregnancy had been regularly monitored by an obstetrician at an external hospital, and no fetal abnormalities were detected during routine prenatal examinations. During early pregnancy, lupus anticoagulant (LA) positivity was identified, and subcutaneous heparin therapy was initiated by her obstetrician. Subsequent immunological evaluation during obstetric follow-up demonstrated anti-*β*2-glycoprotein I antibody positivity. Approximately 2 weeks before admission, repeat LA testing remained positive (LA1/LA ratio, 21.37). Based on these findings, her obstetrician prescribed hydroxychloroquine (200mg daily), aspirin (100 mg daily), and prednisone (30 mg daily), and these medications were continued until admission.

Two days before admission, she developed widespread targetoid erythematous lesions and papulovesicles involving the face, trunk, and extremities, accompanied by tense blisters and mild oral mucosal erosion (Figure 1A). Based on the acute onset of targetoid lesions, blister formation, and mucosal involvement, pregnancy-associated severe erythema multiforme (EM) was initially suspected. Potential triggers of EM were subsequently investigated. Serological testing for herpes simplex virus (HSV)-I/II IgM was positive, whereas Mycoplasma pneumoniae testing was negative; however, Hydroxychloroquine was discontinued immediately after admission because hydroxychloroquine-associated EM has been reported as a potential adverse drug reaction. The medication was not restarted during the subsequent hospitalization or follow-up period. However, considering the relatively long half-life of hydroxychloroquine, residual drug exposure could not be completely excluded. Therefore, although a possible hydroxychloroquine-associated trigger was considered, a definitive causal relationship could not be established.

**Figure 1:**
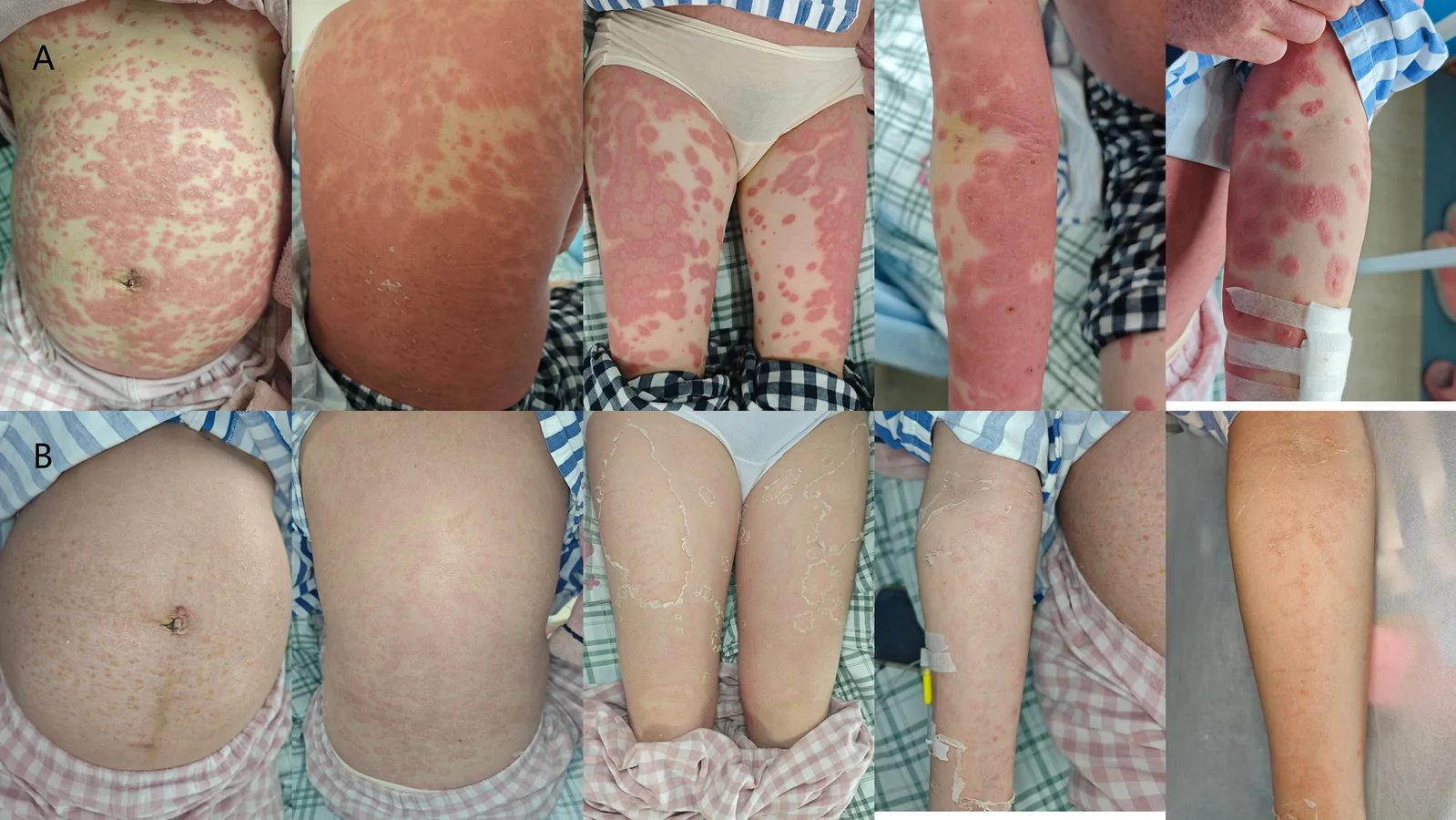
Clinical course and response to certolizumab pegol therapy. **(A)** Baseline clinical presentation showing widespread targetoid erythema, tense blisters, papulovesicular lesions, and pustules involving the trunk and extremities before biologic therapy. **(B)** Marked improvement of cutaneous lesions 72 h after administration of certolizumab pegol, with substantial resolution of erythema, blistering, and pustular lesions.

Potential triggers of EM were subsequently investigated. Serological testing for herpes simplex virus (HSV)-I/II IgM was positive, whereas Mycoplasma pneumoniae testing was negative. However, the positive HSV-I/II IgM result alone was insufficient to establish an active HSV infection or a causal relationship with EM, and no other clinical or laboratory findings suggested a definite infectious trigger. Hydroxychloroquine was discontinued immediately after admission because hydroxychloroquine-associated EM has been reported as a potential adverse drug reaction. The medication was not restarted during the subsequent hospitalization or follow-up period. However, given the relatively long half-life of hydroxychloroquine, residual drug exposure could not be completely excluded. Therefore, although a possible hydroxychloroquine-associated trigger was considered, a definitive causal relationship could not be established. The patient’s complete clinical course and treatment timeline are summarized in Table 1.

**Table 1:** Clinical timeline.

Time point	Gestational age	Clinical events and diagnostic findings	Therapeutic interventions and outcomes
Early pregnancy	First trimester	Routine prenatal follow-up. Lupus anticoagulant positivity was identified during pregnancy monitoring.	Low-molecular-weight heparin was initiated according to obstetric management.
Before admission	30 weeks	Anti-*β*2-glycoprotein I antibody positivity was detected during follow-up evaluation.	Hydroxychloroquine (200 mg/day), aspirin (100 mg/day), and prednisone (30 mg/day) were administered before admission.
28-Jan-25	30 weeks	Developed generalized erythematous papules, papulovesicles, blisters, targetoid lesions, and mild oral erosion for 2 days. Severe EM was initially suspected.	Laboratory evaluation showed marked systemic inflammation. Intravenous methylprednisolone (80 mg/day for 3 days) and IVIG (20 g/day for 5 days) were initiated.
Hospitalization days 2–5	30–31 weeks	Cutaneous lesions progressed despite initial therapy. Sterile pustules developed on erythematous plaques, with pustular coalescence and acral edema, suggesting concomitant PPP.	Skin biopsies were performed from representative lesions.
Early February 2025	Approximately 31 weeks	Histopathology confirmed interface dermatitis with epidermal necrosis compatible with EM and intraepidermal neutrophilic pustules compatible with PPP.	Methylprednisolone pulse therapy (400 mg/day for 3 days) was administered, resulting in partial improvement.
3-Feb-25	Approximately 31 weeks	Persistent erythema and extremity edema indicated incomplete disease control. Genetic testing showed wild-type IL36RN and heterozygous IL36G c.404T > C variant of uncertain significance.	Infection screening was negative. After multidisciplinary evaluation and informed consent, certolizumab pegol (400 mg subcutaneously) was administered.
Within 72 h after certolizumab pegol	Approximately 31 weeks	Rapid improvement of erythema, blisters, pustules, and edema was observed.	Systemic corticosteroids were gradually tapered.
39 weeks + 1 day	Term pregnancy	Pregnancy progressed without recurrence of severe skin disease.	Healthy male infant delivered vaginally. No maternal or neonatal adverse events were observed.
Postpartum follow-up	1 year	Clinical remission maintained.	Continued follow-up without disease recurrence.

### Diagnostic assessment and clinical evolution

Laboratory examination demonstrated systemic inflammation, including leukocytosis (white blood cell count, 20.88 × 10⁹/L), neutrophilia (88.8%), and elevated high-sensitivity C-reactive protein (81.4 mg/L). Hypocalcemia (1.99 mmol/L) was also noted. BP180/BP230 antibodies were negative. Autoimmune evaluation revealed positive antinuclear antibody (ANA) at a titer of 1:100 with a cytoplasmic granular pattern and anti-SSA-52 (Ro52) antibody positivity, whereas other tested autoantibodies, including anti-dsDNA and other extractable nuclear antigen antibodies, were negative. Although lupus anticoagulant and anti-*β*2-glycoprotein I antibodies were detected during pregnancy management, no clinical manifestations fulfilling the diagnostic criteria for systemic lupus erythematosus or antiphospholipid syndrome were identified during hospitalization.

The patient was initially treated with intravenous methylprednisolone (80 mg daily for 3 days) combined with intravenous immunoglobulin (IVIG; 20 g daily for 5 days). However, despite this immunomodulatory therapy, the cutaneous lesions continued to progress. During hospitalization, new sterile pustules developed predominantly on the trunk, with several lesions coalescing into larger pustular plaques. The emergence of pustulation, together with persistent erythema and progressive acral edema, suggested an overlapping pustular psoriatic process during pregnancy (Figure 1A). Given the recent exposure to hydroxychloroquine before disease onset, a drug-related pustular eruption, particularly acute generalized exanthematous pustulosis (AGEP), was also considered as an important differential diagnosis.

Skin biopsies were subsequently performed to further characterize the evolving clinical presentation (Figure 2). Histopathological examination of the arm lesion revealed interface dermatitis with focal epidermal necrosis, findings compatible with erythema multiforme (Figure 2A). In contrast, the abdominal lesion demonstrated intraepidermal pustule formation with prominent neutrophilic infiltration, supporting the diagnosis of pustular psoriasis presenting during pregnancy (PPP) (Figure 2B). Although no prominent eosinophilic infiltration or other specific features suggestive of AGEP were identified, a drug-related pustular reaction could not be completely excluded because drug-specific immunological testing was unavailable. Taken together with the characteristic clinical evolution, these clinicopathological findings supported the diagnosis of concurrent severe erythema multiforme and PPP.

**Figure 2:**
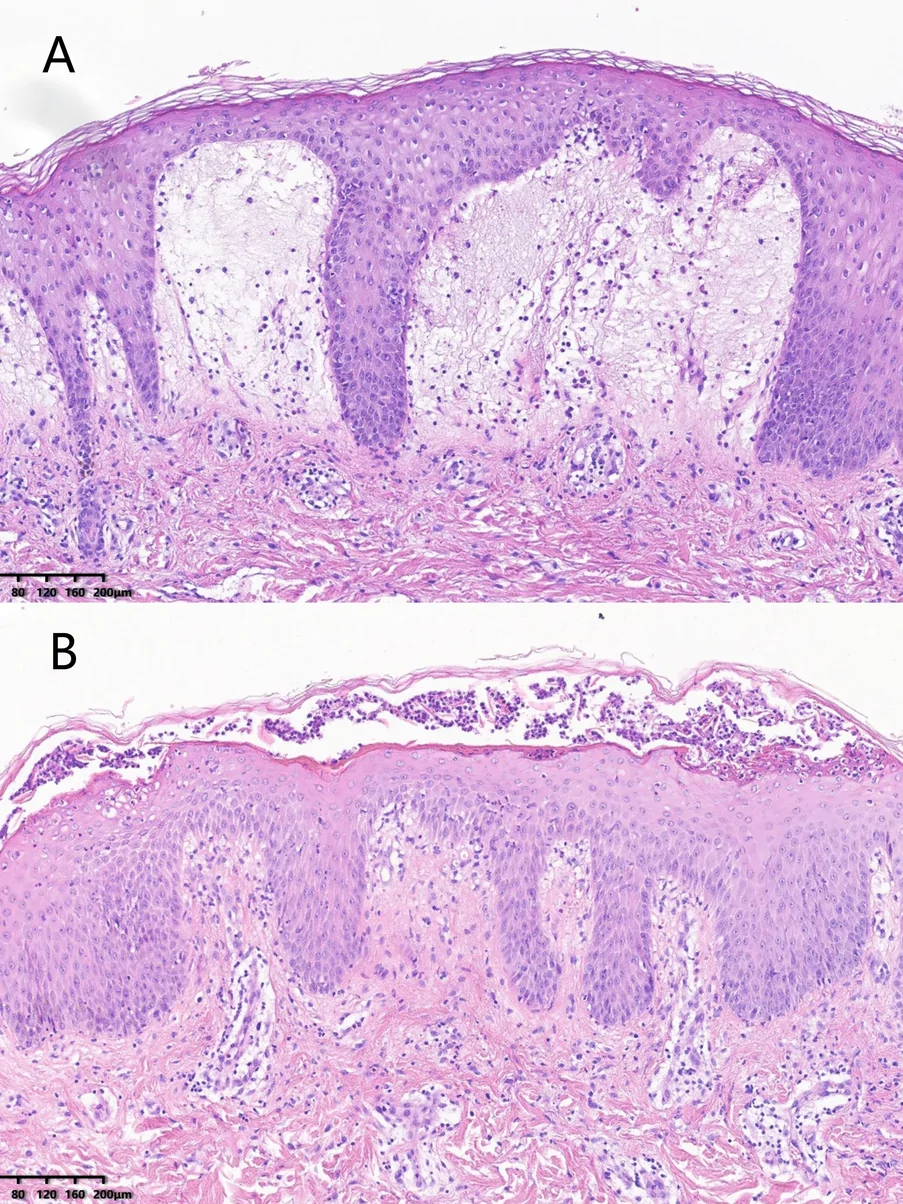
Histopathological findings supporting the diagnosis of concurrent erythema multiforme and pustular psoriasis presenting during pregnancy. **(A)** Histopathological examination of an arm lesion demonstrates interface dermatitis with focal epidermal necrosis, compatible with erythema multiforme. **(B)** Histopathological examination of an abdominal lesion reveals intraepidermal pustule formation with prominent neutrophilic infiltration, supporting the diagnosis of pustular psoriasis presenting during pregnancy.

Because of inadequate response to initial therapy, treatment was escalated to intravenous methylprednisolone pulse therapy (400 mg daily for 3 consecutive days). Although trunk pustulation showed partial improvement, marked erythema and edema of the extremities persisted, indicating ongoing severe inflammatory activity.

Genetic analysis identified wild-type IL36RN and a heterozygous IL36G c.404T > C variant of uncertain significance. Before initiating biologic therapy, screening revealed no evidence of clinically relevant active infections, including tuberculosis and hepatitis B virus infection. Given the refractory disease course, the severity of maternal cutaneous involvement, certolizumab pegol was considered to achieve effective disease control while minimizing prolonged exposure to systemic immunosuppression during pregnancy.

### Therapeutic intervention and outcome

After multidisciplinary discussion and informed consent, a single dose of certolizumab pegol 400 mg was administered subcutaneously. The patient had previously received intravenous methylprednisolone pulse therapy (400 mg daily for 3 consecutive days). On the following day, methylprednisolone was reduced to 200 mg daily, and certolizumab pegol was initiated during the early corticosteroid tapering phase because of persistent severe inflammatory activity. Clinical improvement was observed within 24 h, including reduction of erythema, drying of blisters, and improvement of inflammatory lesions. Marked resolution was evident within 72 h (Figure 1B). Following clinical improvement, methylprednisolone was gradually reduced as follows: methylprednisolone 200 mg for 1 day, 100 mg for 1 day, 80 mg for 4 days,60 mg for 3 days,40 mg for 3 days, after which corticosteroid therapy was discontinued.

The patient was discharged after clinical recovery. She delivered a healthy male infant vaginally at 39 weeks and 1 day of gestation. No congenital abnormalities or maternal/neonatal adverse events related to certolizumab pegol were observed. Disease remission was maintained throughout one year of postpartum follow-up.

## Discussion

This case is clinically notable for the apparent coexistence of severe EM and PPP in late pregnancy. The initial targetoid eruption with mucosal involvement was compatible with severe EM, whereas subsequent sterile pustulation and coalescing pustules, and neutrophilic pustular inflammation on histopathology supported typical PPP. The diagnostic overlap created a therapeutic dilemma because the patient's disease continued to progress despite high-dose systemic corticosteroids and IVIG.

The relationship between EM and PPP remains uncertain. Although both disorders involve immune-mediated inflammation, they represent distinct clinicopathological entities. EM is primarily associated with cytotoxic lymphocyte-mediated keratinocyte injury, whereas PPP involves neutrophil-dominant inflammation with important contributions from the IL-36 pathway and downstream cytokine networks (1, 6, 7). Therefore, this case may represent concurrent inflammatory dermatoses occurring in the setting of pregnancy-associated immune modulation rather than a single shared pathogenic process.

Given the presence of EM-like lesions and positive autoimmune serological findings, Rowell syndrome was considered as a differential diagnosis. However, the patient lacked clinical manifestations fulfilling the criteria for systemic lupus erythematosus, and lupus-associated autoantibodies other than anti-SSA-52 were negative. Therefore, the current findings were insufficient to support a diagnosis of Rowell syndrome. Instead, the clinicopathological features favored severe erythema multiforme occurring in the context of pregnancy-associated immune modulation.

HSV-associated EM was also considered because of the presence of early mucosal involvement and positive HSV-I/II IgM results. However, HSV-I/II IgM positivity alone cannot confirm acute HSV infection because of its limited specificity. Local HSV-DNA testing of mucosal lesions was not performed because no active herpetic lesions suitable for sampling were identified during evaluation. Therefore, although HSV-associated EM could not be completely excluded, no definitive virological evidence supporting an HSV-triggered episode was obtained. In cases with confirmed HSV-associated EM, early antiviral therapy may be beneficial; however, the lack of HSV-DNA confirmation and the absence of typical herpetic manifestations limited the indication for antiviral treatment in this patient.

A possible drug-associated trigger for EM should also be considered. Hydroxychloroquine-associated EM has been reported (8), and in this patient hydroxychloroquine exposure preceded the onset of skin lesions. Hydroxychloroquine was therefore discontinued immediately after admission and was not resumed. However, a definite causal relationship could not be established because of the lack of drug-specific immunological testing and the long elimination half-life of hydroxychloroquine. A lymphocyte stimulation test was not performed because urgent disease control was required during pregnancy and this assay was unavailable at our institution.

The potential influence of corticosteroid therapy should also be considered. High-dose corticosteroids or corticosteroid withdrawal may trigger or aggravate pustular psoriasis (3, 4). In this patient, pustular lesions developed before corticosteroid pulse therapy, and histopathological findings supported PPP. Therefore, corticosteroid exposure alone cannot fully explain the development of pustular lesions. However, the subsequent clinical improvement may have been partially influenced by the cumulative effects of previous immunomodulatory therapies, including corticosteroids and IVIG.

Genetic findings should also be interpreted cautiously. PPP is considered part of the pustular psoriasis spectrum, in which IL36RN mutations have been identified in a subset of patients (3, 6, 7). Genetic testing was performed to explore potential molecular contributors in this severe and refractory case. However, IL36RN was wild-type, and the identified heterozygous IL36G c.404T > C variant remains a variant of uncertain significance. This finding should be considered hypothesis-generating only, and further functional and population-level data would be required before assigning pathogenic significance (4, 6, 7).

Recent advances in the understanding of IL-36-mediated inflammation have led to the development of targeted therapies, including IL-36 receptor inhibition with spesolimab, which has demonstrated efficacy in generalized pustular psoriasis flares (9). In the randomized Effisayil 1 trial, 54% of patients treated with spesolimab achieved a GPPGA pustulation subscore of 0 at Week 1, compared with 6% of those receiving placebo (9). Similarly, the phase II GALLOP study of the IL-36 receptor inhibitor imsidolimab reported clinical responses as early as Day 3, with pustulation showing particularly early improvement (10). In the present case, clinical improvement was observed within 24 h after administration of certolizumab pegol, with marked improvement within 72 h. Although these observations cannot be interpreted as a direct comparison of efficacy because of differences in study populations, disease phenotypes, treatment regimens, and outcome measures, the rapid clinical response observed with certolizumab pegol was within a similar temporal range to the early responses reported with IL-36-targeted therapies. However, evidence regarding the safety and efficacy of IL-36-targeted therapies during pregnancy remains unavailable, limiting their current application in pregnant patients. In this context, biologic agents with established pregnancy safety profiles may represent more practical therapeutic options for severe and refractory inflammatory dermatoses during pregnancy.

Certolizumab pegol was selected based on both immunological rationale and pregnancy safety considerations. In this patient, certolizumab pegol was not used as initial therapy but was considered after inadequate response to systemic corticosteroids, IVIG, and subsequent methylprednisolone pulse therapy. Because persistent inflammatory activity remained despite intensive immunomodulatory treatment, and prolonged exposure to high-dose corticosteroids during pregnancy was undesirable, certolizumab pegol was administered as an individualized rescue strategy.

TNF-α participates in inflammatory pathways involved in both EM and pustular psoriasis. In EM, TNF-α may participate in keratinocyte apoptosis and drug-related epithelial injury; in pustular psoriasis, TNF-α may amplify neutrophil-rich cytokine networks alongside IL-36, IL-1, IL-6, and IL-17/IL-23 pathways (1, 6, 7). This shared inflammatory background provides a plausible mechanistic rationale for TNF-α blockade in selected refractory cases.

 (4)

Biologic therapies are increasingly considered for severe immune-mediated diseases during pregnancy when conventional treatments are insufficient or prolonged immunosuppression may pose additional risks. TNF-α inhibitors have the most extensive pregnancy safety experience among biologic agents (11, 12). Although evidence supporting TNF inhibition in PPP remains limited, several reports have described TNF inhibitor use in pregnancy-associated pustular psoriasis or generalized pustular psoriasis (3, 4). However, TNF inhibition is not currently standard therapy for PPP. In this case, certolizumab pegol was used as an individualized rescue therapy because of persistent disease activity despite corticosteroids, IVIG, and corticosteroid pulse therapy.

Compared with other TNF inhibitors, certolizumab pegol lacks an Fc region and therefore has minimal FcRn-mediated placental transfer. The CRIB study demonstrated minimal placental transfer during late pregnancy, while the CRADLE study showed minimal transfer into breast milk (5, 13). These characteristics support its consideration when biologic therapy is required during pregnancy. Nevertheless, TNF inhibitors may rarely induce paradoxical inflammatory reactions, including psoriasiform eruptions and EM-like reactions (14, 15). Therefore, the potential benefits and risks of biologic therapy should be carefully balanced through multidisciplinary evaluation.

Although marked improvement occurred shortly after certolizumab pegol administration, the contribution of previous therapies cannot be excluded. In this case, certolizumab pegol was administered on the day following completion of methylprednisolone pulse therapy (400 mg daily for 3 consecutive days), while corticosteroids were being tapered. Therefore, certolizumab pegol was introduced during a period of ongoing immunomodulatory treatment rather than as an isolated intervention.

The close temporal association between certolizumab pegol administration and clinical improvement suggests a potential therapeutic contribution of TNF-α blockade; however, the rapid response cannot be definitively attributed to certolizumab alone (5, 11). Therefore, clinical improvement likely reflected the combined effects of sequential immunomodulatory treatments, including corticosteroids, IVIG, and TNF-α inhibition.

Several limitations should be acknowledged. First, as a single case report, this study cannot establish a causal relationship between certolizumab pegol administration and clinical improvement. Second, previous corticosteroid and IVIG therapy may have contributed to disease resolution. Third, long-term infant outcomes, including neurodevelopmental and immune function, were not systematically assessed.

Despite these limitations, this case provides additional clinical experience regarding biologic rescue therapy for severe inflammatory dermatoses during pregnancy. Larger case series and registry-based studies are needed to clarify the efficacy, safety, optimal timing, and clinical positioning of biologic therapies in this setting.

## Conclusion

Severe EM associated withPPP during pregnancy represents a challenging clinical scenario requiring individualized management to balance maternal disease control and fetal safety. In this case, certolizumab pegol was associated with rapid clinical improvement following inadequate responses to corticosteroids and IVIG, with no maternal or neonatal adverse events. Although the contribution of previous therapies cannot be excluded, certolizumab pegol may represent a potential rescue option for selected patients with severe refractory cases because of its inflammatory targeting rationale and favorable pregnancy safety profile. Further clinical experience and registry-based studies are required to define optimal treatment strategies.

## Patient perspective

The patient expressed satisfaction with the rapid improvement of her skin condition and the successful continuation of pregnancy. She agreed that her clinical experience may contribute to improving understanding of treatment options for similar patients.

## Data Availability

The datasets presented in this study can be found in online repositories. The names of the repository/repositories and accession number(s) can be found in the article/Supplementary Material.
